# Evaluating COVID-19-Related Disruptions to Effective Malaria Case Management in 2020–2021 and Its Potential Effects on Malaria Burden in Sub-Saharan Africa

**DOI:** 10.3390/tropicalmed8040216

**Published:** 2023-04-04

**Authors:** Paulina A. Dzianach, Susan F. Rumisha, Jailos Lubinda, Adam Saddler, Mauricio van den Berg, Yalemzewod A. Gelaw, Joseph R. Harris, Annie J. Browne, Francesca Sanna, Jennifer A. Rozier, Beatriz Galatas, Laura F. Anderson, Camilo A. Vargas-Ruiz, Ewan Cameron, Peter W. Gething, Daniel J. Weiss

**Affiliations:** 1 Child Health Analytics, Telethon Kids Institute, Nedlands, WA 6009, Australia; 2 Strategic Information for Response, Global Malaria Programme, World Health Organization, 1211 Geneva, Switzerland; 3 Faculty of Health Sciences, Curtin University, Perth, WA 6102, Australia

**Keywords:** malaria, COVID-19, effective treatment

## Abstract

The COVID-19 pandemic has led to far-reaching disruptions to health systems, including preventative and curative services for malaria. The aim of this study was to estimate the magnitude of disruptions in malaria case management in sub-Saharan Africa and their impact on malaria burden during the COVID-19 pandemic. We used survey data collected by the World Health Organization, in which individual country stakeholders reported on the extent of disruptions to malaria diagnosis and treatment. The relative disruption values were then applied to estimates of antimalarial treatment rates and used as inputs to an established spatiotemporal Bayesian geostatistical framework to generate annual malaria burden estimates with case management disruptions. This enabled an estimation of the additional malaria burden attributable to pandemic-related impacts on treatment rates in 2020 and 2021. Our analysis found that disruptions in access to antimalarial treatment in sub-Saharan Africa likely resulted in approximately 5.9 (4.4–7.2 95% CI) million more malaria cases and 76 (20–132) thousand additional deaths in the 2020–2021 period within the study region, equivalent to approximately 1.2% (0.3–2.1 95% CI) greater clinical incidence of malaria and 8.1% (2.1–14.1 95% CI) greater malaria mortality than expected in the absence of the disruptions to malaria case management. The available evidence suggests that access to antimalarials was disrupted to a significant degree and should be considered an area of focus to avoid further escalations in malaria morbidity and mortality. The results from this analysis were used to estimate cases and deaths in the World Malaria Report 2022 during the pandemic years.

## 1. Introduction

The COVID-19 pandemic wreaked havoc globally, both in terms of the direct death toll and the indirect effects on healthcare delivery, the global economy, individuals’ mobility, and the general well-being of populations worldwide. In Africa, as elsewhere, impacts on the prevention and treatment of other diseases were likely to have been important, but the magnitude of effects on morbidity and mortality remain poorly understood. Malaria is of particular concern in Africa given the high burden of the disease in sub-Saharan countries. In the year prior to the onset of the pandemic (2019), there were an estimated 232 million (213–255) malaria cases and 568 thousand (532–654) deaths globally, with around 96% of all cases and 97% of all deaths occurring within the African continent [1].

In the early stages of the pandemic, a modelling exercise was undertaken to evaluate the hypothetical effects of disruptions to malaria interventions on morbidity and mortality in Africa [2]. That study suggested that if core malaria control interventions—indoor residual spraying (IRS), insecticide-treated nets (ITNs) and antimalarial treatment (AM)—were to be substantially reduced as a result of the pandemic, malaria mortality could almost double in 2020 [2]. Recognition of this threat [3] led to a concerted effort to overcome barriers to the implementation of vector control, and data obtained for 2020 and 2021 show that most ITN and IRS campaigns scheduled were completed despite the immense challenges, albeit with a large proportion of campaigns experiencing some delays [1,4].

In contrast to campaign-oriented vector control delivery, access to antimalarial treatment relies on both a functioning routine health system and population behaviour, and there is a general consensus that substantial disruptions to malaria case management occurred as a result of the pandemic. The extent and impact of these disruptions, however, remains unclear, and the challenge of quantifying their magnitude is exacerbated by the impact that the pandemic had on the availability and quality of routinely collected malaria data.

An annual exercise is undertaken to estimate the malaria burden in Africa to inform WHO reporting [5] and Global Burden of Disease studies [2,6,7], as well as other global stakeholders. In this study, we describe the extension of the established burden estimation framework to account for the impact of disrupted malaria case management on the malaria burden in Africa during the COVID-19 pandemic. Along with environmental data and information on vector control coverage readily available for the pandemic period, the existing modelling framework for estimating annual malaria burden in the moderate-to-high-burden countries of sub-Saharan Africa relies on the data obtained from Demographic and Health Surveys (DHS) and Malaria Indicative Surveys (MIS) [8]. These surveys are a key resource for data on both malaria infection prevalence and the population’s access to effective antimalarial treatment. However, surveys for a given country are often spaced years apart, and this cadence was slowed further due to the pandemic, with many national surveys being postponed [9]. As such, this indirect effect of the pandemic limited our ability to include empirical data that captured changes in either malaria or malaria treatment.

In this context, the aims of this research were to (a) obtain plausible estimates of the magnitude of COVID-19-related impacts on access to antimalarial treatment in the 2020–2021 period, and (b) to develop estimates of changes in malaria burden that reflect these impacts.

## 2. Materials and Methods

### 2.1. Overview

For the first of our aims, we used country-level disruption estimates collected by the WHO in the three rounds of the “Global pulse survey on continuity of essential health services during the COVID-19 pandemic” [10,11,12], hereafter referred to as the Pulse surveys. We used the survey responses to estimate the thresholds of disruptions to malaria diagnosis and treatment. Next, we updated an established spatiotemporal Bayesian geostatistical model [2,13] to produce estimates of malaria’s clinical incidence and mortality that enumerated antimalarial treatment rates with and without disruptions. We compared these results to quantify the effect of possible malaria case management disruptions on malaria incidence and mortality for 2020–2021. The resulting estimates of malaria incidence, adjusted for pandemic-related treatment disruptions, were ultimately used for the 2022 World Malaria Report (WMR) [1,4].

### 2.2. Geographic Scope

This study focuses on the 32 sub-Saharan African countries carrying the highest burden of malaria, for which established geospatial models used for estimating the *Plasmodium falciparum* parasite rate (*Pf*PR) [14], clinical incidence [15], and mortality [13,16] incorporate the role of effective malaria treatment in mediating the burden. Collectively, these 32 countries contributed an estimated 97.9% of malaria cases in sub-Saharan Africa and 93.6% of malaria cases globally in 2019 [1].

### 2.3. WHO Pulse Surveys

The Pulse survey data consisted of estimated service disruptions to all healthcare services in response to the pandemic, as reported by public health officials via a structured questionnaire. While based on in-country expert opinion, and thus inherently subjective, the Pulse survey data were nonetheless chosen as the most informative available data source for capturing the magnitude of disruptions to malaria case management and, thus, was incorporated in the adjusted model of malaria burden. This decision was based on (a) the geographic extent of the survey, which included responses from all of the 32 countries; (b) the between-country comparability of the data, as the questions were standardized across countries; (c) the repeat nature of the survey, which provided three estimates of service disruptions spanning the pandemic period for which major disruptions occurred; and (d) the survey design that avoided biases of rapid phone- or social-media-based surveys that tend to oversample wealthier and more urban demographic groups [17]. An analysis of patient records (such as tracking the number of patients attending health facilities) was also considered as a quantitative basis for evaluating disruptions without reliance on expert opinion. However, the sources identified [18,19,20] did not cover the whole study region or span the full study period. Further, patient records cannot on their own reflect the changes in effective case management for malaria. For instance, if the numbers of patients on record increase, it does not necessarily mean that a higher proportion of cases is being effectively treated for malaria—it could also indicate a malaria outbreak or reduced access to antimalarials in that region. As information which would be necessary to untangle these confounding factors from the patient records was lacking, it was decided that expert opinion on the disruptions in malaria case management was the best available data source for our analysis.

**Figure 1:**
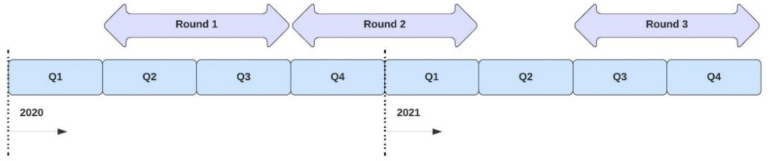
Time coverage of the Pulse surveys.

The first Pulse survey round was completed from May to September 2020, the second round from January to March 2021, and the third round from November to December 2021 [10,11,12]. The respondents were asked to answer questions on disruptions experienced in each health service included in the questionnaire in the last three months for the first and second survey rounds and for the last six months in the third survey round. For the first survey, the responses were assumed to reflect the key informants’ view on the situation in their country in the second and third quarters of 2020. The second survey was considered representative for the fourth quarter of the year 2020 and the first quarter of 2021. For the third survey, the responses were assumed to be representative of the third and fourth quarters of 2021 (see Figure 1).

The survey questionnaires varied between the three rounds as follows. In the first round, questions were asked about the disruption in malaria diagnosis and treatment using a three-point ordinal scale (<5%; 5–50%; >50% of patients not served as usual). In contrast, in the second and third rounds, a four-point ordinal scale was used (<5%; 5–25%; 26–50%; >50% of patients not served as usual). Respondents could also respond “Do not know” or “Not applicable”, and these data points were excluded from our analysis.

### 2.4. Calculating Upper and Lower Disruption Thresholds—WHO Pulse Survey

An initial step was required to convert the available disruption data from the three Pulse survey rounds into an inferred complete disruption time series, with upper and lower bounds for each country for the years 2020–2021. During each survey round, each respondent was asked for an estimate on disruptions in the preceding three or six months, and we attributed each response to specific quarters of the year as follows. The first quarter of the year 2020 (January–March 2020) was assumed to have had no COVID-19-related disruptions, as the pandemic state was declared only in March 2020. For the second quarter and third quarter (April–September 2020), we assigned the disruption intervals from the first round of the survey for each country. For the fourth quarter of 2020 and first quarter of 2021 (October 2020–March 2021), we assigned the disruption intervals from the second round of the survey. For the third and fourth quarters of the year 2021, we assigned the disruption intervals from the third round of the survey. For the second quarter of 2021, as the surveys did not cover this period, we used the minimum estimate from the two rounds in 2021 (i.e., round two and round three) as the lower limit and the maximum estimate from these two rounds as the upper limit. In the rare instances when the survey respondent reported that more than 50% of users were not served as usual, we set both the upper and lower limits of disruptions to 50% for that round.

Let *r*_1_(*i*), *R*_1_(*i*) denote the lower and upper limits, respectively, of disruptions reported for country *i* in round one of the Pulse survey. Analogously, let *r*_2_(*i*), *R*_2_(*i*) denote the lower and upper limits of disruptions for country *i* in round two of the survey, etc. Finally, let *δ_low_*(*i*), *δ_high_*(*i*) denote the lower and upper limits of annual disruption values for country *i*, respectively. Assuming that survey responses were received from country *i* for two survey rounds, the annual disruptions to antimalarial treatment rates for the years 2020–2021 were calculated as follows:

(1) $$\delta_{low_{2020}}\left(i\right)=\frac{1}{4}\left(2r_{1}\left(i\right)+r_{2}\left(i\right)\right),$$

(2) $$\delta_{low_{2021}}\left(i\right)=\frac{1}{4}\left(r_{2}\left(i\right)+\min\left(r_{2}\left(i\right),r_{3}\left(i\right)\right)+2r_{3}\left(i\right)\right),$$

(3) $$\delta_{high_{2020}}\left(i\right)=\frac{1}{4}\left(2R_{1}\left(i\right)+R_{2}\left(i\right)\right),$$

(4) $$\delta_{high_{2021}}\left(i\right)=\frac{1}{4}\left(R_{2}\left(i\right)+\max\left(R_{2}\left(i\right),R_{3}\left(i\right)\right)+2R_{3}\left(i\right)\right).$$

A shortcoming of the Pulse surveys is that responses were not received from all countries in each round, which necessitated approximating the missing round(s) using the available responses. If a given country responded in only one survey round, these responses were applied to the other survey round periods. For example, if country *i* only responded in survey round one, then we set *r*_3_(*i*) = *r*_2_(*i*) = *r*_1_(*i*) and *R*_3_(*i*) = *R*_2_(*i*) = *R*_1_(*i*) and applied the above equations. If a given country responded to two rounds, our approach differed depending on the round that was missing. If rounds one or three were missing, we set the missing value to that of round two as it was closest in time. If round two was missing, we followed the same logic used for approximating Q2 of 2021 by setting the lower estimate to the minimum value of surveys one and three and the upper estimate to the maximum value of surveys one and three.

### 2.5. Estimating Malaria Case Incidence and Mortality

Our modelling framework for assessing the impacts on malaria from changing intervention coverages has been described previously [2,7,21]. In brief, the approach consists of (i) a spatiotemporal Bayesian geostatistical model for predicting *Pf*PR, with terms capturing effects for ITNs, IRS, and AMs; (ii) a natural history model that predicts clinical incidence rate as a function of *Pf*PR and which also includes terms for the effects on this relationship of AM treatment; and (iii) an established geospatial model to predict malaria-attributable mortality given the incidence rate and effective treatment rates, calibrated to malaria-specific and an all-cause mortality envelope provided by the Global Burden of Disease study [22].

We applied this framework to estimate the impact of COVID-19 disruptions to AM treatment on malaria case incidence as follows. First, a baseline *Pf*PR model was run in which AM treatment coverage was set to levels estimated for 2020 in the absence of disruptions, generating 100 pixel-level realizations of population-weighted prevalence estimates for each of the 32 countries for the years 2020 and 2021. We then reran the *Pf*PR model configured with AM coverages reflecting the disruption ranges reported in the Pulse surveys, thereby generating for each country a second set of 100 *Pf*PR realizations per modelled year, for which the relative decrease in AM coverage was uniformly distributed in 5% increments within the country’s disruption range. For instance, if the disruption range for a given year was 10–25%, then a set of 25 realizations was taken from modelled outputs where AM coverage was reduced by 10%, 15%, 20%, and 25% to create a full set of 100 *Pf*PR realizations with disruptions. Both baseline and disrupted *Pf*PR predictions were converted to malaria clinical incidence [13,15], and mortality was estimated by applying previously established predictions of the case fatality rate of untreated malaria (uCFR) [13,16] to the baseline and disrupted incidence predictions, incorporating the AM coverage disruptions into estimates of malaria mortality.

## 3. Results

### 3.1. Estimating Malaria Case Incidence and Mortality

**Figure 2:**
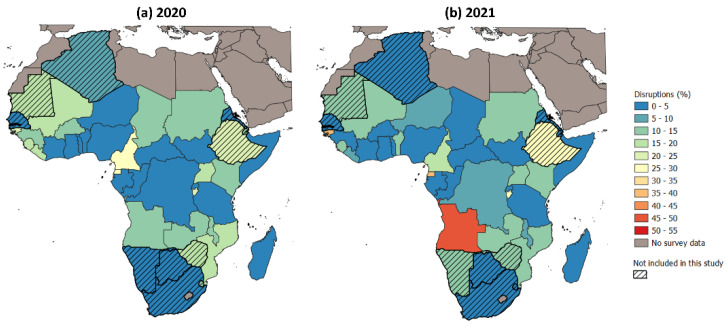
Estimated national-level disruptions in malaria case management in (**a**) 2020 and (**b**) 2021.

Figure 2 illustrates the estimated mean annual disruptions to effective antimalarial treatment derived from the Pulse survey rounds and our approaches for estimating un-surveyed periods and missing survey values. In 2020, the annual national-level disruptions, as averaged across all four quarters and both upper and lower estimates, ranged from 1.9 to 28.5% of individuals seeking healthcare for malaria not being served as usual, whereas in 2021, this range was 2.5 to 50.0%. The majority (21/32) of countries in the study region experienced mean annual disruptions of larger magnitude in 2021 compared with 2020, which is consistent with the fact that the COVID-19 pandemic was only declared at the end of the first quarter of 2020. When we look at the successive responses, however, we observed that the proportion of countries reporting significant disruptions to malaria treatment decreased as the pandemic progressed (Table 1). In particular, in round one, 12/21 countries in the study region responded that the disruptions were more than 5%. In round two, 9/21 countries responded that they experienced disruptions of over 5%. Finally, in round three, only 4/23 countries in the study region reported disruptions of more than 5%.

Although the proportion of countries reporting significant disruptions to malaria diagnostics and treatment reduced as the pandemic progressed, it should be noted that disruptions of higher that 50% were reported only in round two. This magnitude of disruptions was reported by Angola and Cameroon, signifying that this was an especially high period of disruptions in these countries.

The countries in sub-Saharan Africa estimated to have experienced the greatest disruptions in 2020 were Equatorial Guinea (28.5%), Cameroon (26.3%), and Guinea-Bissau (23.3%). In 2021, the highest disruptions were estimated for Angola (50.0%), Equatorial Guinea (38.0%), and Guinea-Bissau (38.0%), along with Ethiopia (27.5%) and Burundi (27.5%).

### 3.2. Effect of Disruptions to Malaria Case Management on Malaria Case Incidence in 2020–2021

**Figure 3:**
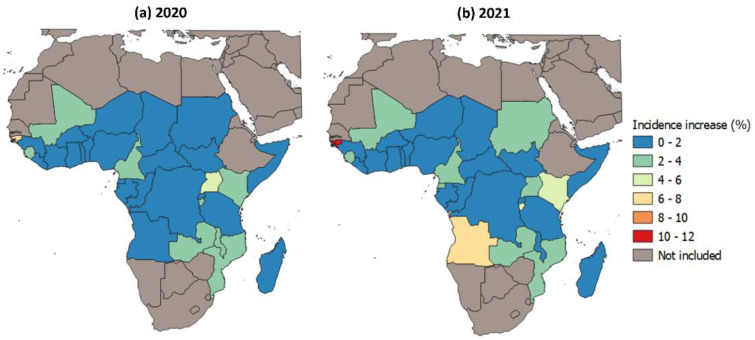
Estimated national level % mean increase in malaria incidence in the study region brought upon by disruptions in malaria case management in (**a**) 2020 and (**b**) 2021.

A reduction in antimalarial treatment coverage is expected to increase malaria transmission by lengthening the time in which people with malaria parasites remain infectious in the community. The estimated increase in malaria incidence brought upon by the disruptions in malaria case management is illustrated in Figure 3. In 2020, we estimated that the mean national level of malaria incidence increases were in the range of 0.1–6.3%. Across the study region as a whole, the number of malaria cases increased by 1.2% (0.3–2.1 95% CI) in 2020 and 1.2% (0.4 to 2.1 95% CI) in 2021, which translated to approximately 2.9 (2.2–3.5 95% CI) million and 3.0 (2.2–3.7 95% CI) million more malaria cases in 2020 and 2021, respectively, compared to a scenario without disruptions to malaria case management. The countries with the greatest increase in case estimates were Guinea-Bissau (6.3%; 95% CI 1.6–10.9%) and Uganda (4.8%; 1.2–7.4%) in 2020. However, in 2020, mean increases less than 1% were predicted for 15/32 countries (Table S1).

In 2021, the mean national level increase in malaria cases ranged from 0.1 to 10.2%. In this period, the highest increases in incidence brought upon by the disruptions were estimated again in Guinea-Bissau (10.2%; 5.4–15.3%) and in Angola (6.9%; 5.3–9.0%). In total, only 3/32 counties (Guinea-Bissau, Angola, and Burundi) were estimated to have experienced a mean increase in cases larger than 5% due to disruptions to effective treatment. Conversely, 18/32 countries were predicted to experience mean malaria case load increases due to the disruptions of less than 1% in 2021, and 19/32 countries experienced disruption-attributable increases in malaria cases of less than 2% throughout the 2020–2021 study period. Consistent with the Pulse survey estimates shown in Figure 2, most of countries experienced milder disruptions in 2021 compared to 2020.

### 3.3. Effect of Disruptions to Malaria Case Management on Malaria Mortality in 2020–2021

**Figure 4:**
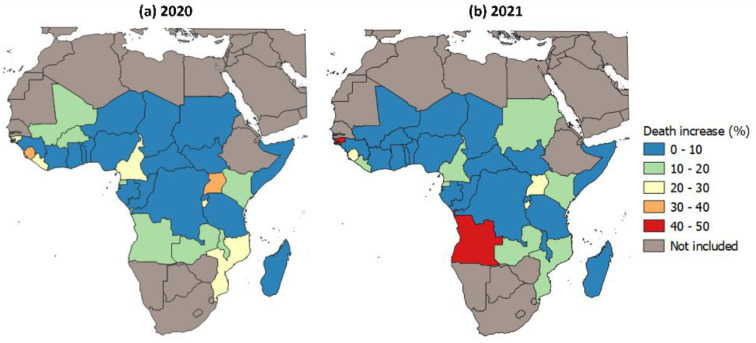
Estimated national level % mean increase in malaria mortality in the study region brought upon by disruptions in malaria case management in (**a**) 2020 and (**b**) 2021.

The modelling framework recognizes that reduced case management impacts malaria mortality via two pathways: by increasing the overall number of malaria cases via effects on transmission and by increasing the fraction of those cases that do not receive prompt and effective treatment and, hence, are more likely to progress to severe disease and death. As such, the proportional increase in malaria mortality was expected to be greater than in case incidence. Overall, we estimated that disruptions to malaria case management caused 38 (8–68 95% CI) thousand more malaria deaths in 2020 as opposed to the counterfactual scenario in which there were no disruptions to effective treatment. Results for 2021 were similar, with an estimated 38 (12–64.0 95% CI) thousand additional deaths. Figure 4 illustrates the estimated increase in malaria deaths at the national level. Mirroring the pattern observed in incidence, most countries had increases in deaths at or below 10%, although estimates for some countries were much higher. Of particular concern here are Angola and Guinea-Bissau in 2021, for which the estimated increases in deaths were 47.6% (45.3–50.2 95% CI) and 44.6% (32–55.0 95% CI), respectively.

**Figure 5:**
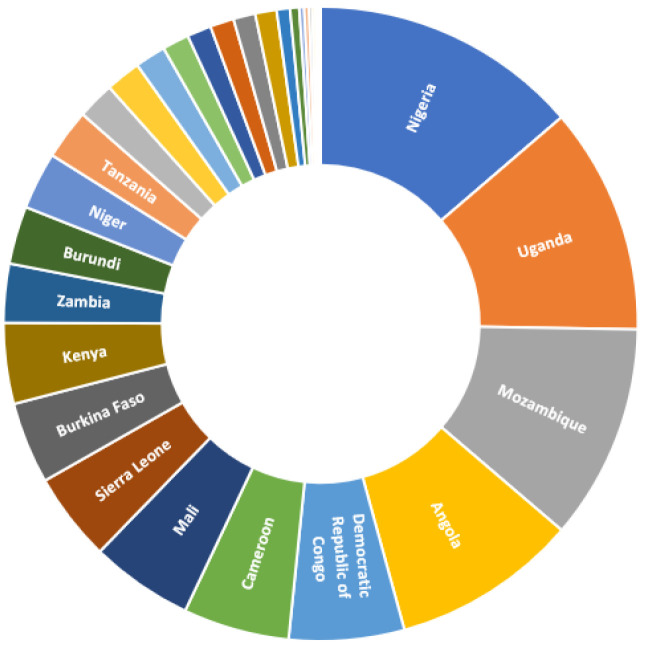
Distribution of additional malaria deaths per country in the 2020–2021 period.

In terms of absolute increases in malaria mortality, Nigeria, Uganda, Mozambique, and Angola were expected to have been most affected during the 2020–2021 period, with a combined proportion of around 46% of additional malaria deaths expected to have occurred in these countries (Figure 5). This largely reflects their large baseline contribution to absolute malaria mortality in undisrupted years.

## 4. Discussion

The COVID-19 pandemic posed an enormous challenge for health systems worldwide, both in terms of managing the new disease and maintaining access to existing healthcare services. The indirect impacts of COVID-19 on healthcare include inhibited movement, scaling down operations by health facilities, increased cost of care, avoidance of health facilities due to fear of COVID-19, the diversion of resources to manage COVID-19, and supply shortages (such as PPE equipment or medicine)—see Table 2.

Some areas of the health systems in the sub-Saharan region exhibited considerable resilience in the face of this healthcare crisis, as demonstrated by the completion of most scheduled ITN and IRS campaigns in 2020 [4] and by reports of increased access to HIV services in 2020 in comparison with previous years across the region [53,54,55]. Nonetheless, while steps were taken to maintain healthcare services for essential ailments, the scope of the pandemic rendered some level of disruption to healthcare provision and utilization unavoidable.

This analysis of 32 high-malaria-burden sub-Saharan countries suggests that disruptions in access to antimalarial treatment resulted in an increase in malaria cases of just over 1% in both 2020 and 2021, compared with baseline conditions, which represents around 5.9 million more malaria cases in the two-year period. These impacts were not equally distributed, however, with some countries likely to have had increases in excess of 5%.

Our earlier analysis estimated that a 25% reduction in antimalarial drug coverage would be expected to lead to a disastrous 26% increase in malaria deaths [2]. The analysis presented here suggests that the extent of disruptions to malaria treatment was considerably lower than this scenario, and, accordingly, the worst potential impacts were avoided. Nonetheless, we still estimated that the number of malaria deaths in the 2020–2021 period was approximately 8% higher than it would have been if no disruptions to treatment occurred. Furthermore, although the proportional impacts were concentrated in only a few countries with increases in mortality of up to 40%, the absolute impact on deaths was greatest in highly malaria-endemic countries with larger populations that had relatively modest levels of disruption. Our estimates on the additional number of malaria deaths that occurred during the pandemic differ from those reported by the World Health Organization (76 thousand additional deaths in the study region versus 63 thousand additional deaths globally, reported in WMR2022 [1]) due to a difference in the methodologies used to estimate deaths in the presence and absence of AM treatment disruptions and the one used in this analysis. First, in the WMR2022, the relative increase in deaths that resulted from this analysis was applied to the estimates of mortality in children under five years old, to obtain the additional number of malaria deaths in this age group as a result of the COVID-19-related disruptions. These estimates were transformed into all-age mortality using a pre-established relationship between the two age groups (more details on this method can be found on page 135 in WMR2022, under the subheading of ‘Category 2 method’). On the other hand, our estimates were calculated directly on the all-age mortality trends. Second, the global number reported by the WHO includes additional deaths due to COVID-19 estimated for India (outside of this study’s region), for which a separate approach to that presented in this analysis was used (more details on this method can be found on page 133 in WMR2022).

An important limitation of the approach proposed here is its reliance on expert opinion for quantifying the extent of disruptions to malaria case management. Although the Pulse surveys identified qualified experts with great familiarity of local health systems, the true extent of disruptions across each health system is very difficult to estimate accurately because it was influenced by complex and poorly documented phenomena including staff shortages, malaria commodity stockouts, or price increases [56]. An additional consideration is potential shifts in patient behaviour towards accessing treatment through alternative means outside of health facilities, such as through community posts [57] or pharmacies [56], in response to fears of becoming infected with the new virus. Even if such complicating factors were knowable, they may or may not have been captured in the reported disruption data based on the interpretation of the question by the expert respondent. Another limitation was the incomplete temporal record of the data, as the Pulse surveys lacked estimates representative of the second quarter of 2021, and not all countries had responses for each of the three survey rounds. This could in some cases result in the overestimation or underestimation of disruptions for the year quarters with missing data. The most notable example here is the high estimated disruptions in Angola for 2021, which came from a report of “More than 50%” disruptions in round two of the Pulse surveys. These disruptions were applied to all four quarters of the year 2021, due to missing data for Angola in round three; however, it is possible that in reality, the country experienced smaller disruptions later that year. Lastly, our analysis did not consider reduced human mobility (including international movements) and its role in malaria transmission, seasonal malaria chemoprevention campaigns, novel malaria mitigation measures that could have been introduced in response to the pandemic, or heterogeneity within countries with regard to disruptions in antimalarial treatment.

The COVID-19 pandemic has led to far-reaching disruptions to healthcare provision and utilization. Although many countries in the sub-Saharan region demonstrated remarkable resilience in the face of the global pandemic, the disruptions to healthcare provision and utilization had a notable impact on malaria case incidence and an even greater impact on malaria mortality. Although access to malaria-preventative commodities such as insecticide-treated nets or indoor residual spraying were mostly maintained, effective treatment for malaria must be brought back to at least pre-pandemic levels to resume global progress against malaria morbidity and mortality.

## Figures and Tables

**Table 1:** Number of countries in the study region reporting disruptions within the specified magnitude brackets.

	<5%	5–50%	>50%
Round one	9	12	0
Round two	12	7	2
Round three	19	4	0

**Table 2:** Indirect impacts of COVID-19 on healthcare services.

**Indirect impacts of COVID-19 on healthcare services**	**Inhibited movement**	**Increased cost of transport** [23,24,25]
		**Suspension of public transport** [26,27,28]
		**Government-imposed movement restrictions** [29,30,31]
	**Scaling down operations by health facilities**	**Temporarily closing down** [32,33,34]
		**Shortening opening hours** [35,36,37]
		**Limiting operations to essential services** [38,39,40]
	**Increased cost of care**	**Direct increase in costs due to additional cost of PPE and increased price of medicine** [27,32,41]
		**Perceived increase in the costs due to loss of income** [42,43,44]
	**Other**	**Avoiding health facilities due to fear of COVID-19** [45,46,47]
		**Repurposing resources to manage COVID-19** [48,49,50]
		**Supply shortages (PPE equipment and medicine)** [36,51,52]

## Data Availability

The data used in this analysis may be shared upon request by contacting the Malaria Atlas Project (malariaatlas@telethonkids.org.au), subject to data sharing agreements with third parties.

## Supplementary Materials

The following supporting information can be downloaded at: https://www.mdpi.com/article/10.3390/tropicalmed8040216/s1, Table S1: Country-level disruptions in malaria effective treatment, and the subsequent malaria burden increase.
